# Application of online teaching mode combining case studies and the MOOC platform in obstetrics and gynecology probation teaching

**DOI:** 10.1186/s12909-022-03854-1

**Published:** 2022-11-17

**Authors:** Yi Li, Chunfen Yang, Wenyan Liao, Shuangjian Yang, Wenjuan Tong, Li Li, Hui Lan, Dong Yang

**Affiliations:** 1 The First Affiliated Hospital, Department of Obstetrics and Gynecology, Hengyang Medical School, University of South China, No. 69, Chuanshan Road, Hengyang, 421001 Hunan China; 2 The Hospital Management Institute of University of South China, Hengyang, 421001 Hunan China; 3 Department of Obstetrics and Gynecology, The First Affiliated Hospital of Guangxi Medical University, No. 6, Shuangyong Road, Nanning, 530021 Guangxi China; 4 The First Affiliated Hospital, Department of Oncology, Hengyang Medical School, University of South China, No. 69, Chuanshan Road, Hengyang, 421001 Hunan China; 5 Department of Radiation Oncology, the First Affiliated Hospital of Guangxi Medical University, No. 6, Shuangyong Road, Nanning, 530021 Guangxi China

**Keywords:** Case studies teaching, Clinical basic integration teaching mode, MOOC platform, Obstetrics and gynecology internship teaching, Online teaching mode, Teaching effect

## Abstract

**Objective:**

To explore the application effect of the clinical basic integration teaching mode constructed by case studies and the MOOC platform in obstetrics and gynecology internship teaching in the face of public health emergencies.

**Methods:**

One hundred ten clinical medical students of grade 2020 were selected as the experimental group, and 110 clinical medical students of grade 2021 were selected as the control group. The experimental group adopted the online teaching mode combined with case studies and the MOOC platform, while the control group adopted the offline traditional probation teaching method. Comprehensive test and questionnaire were used to evaluate and compare the teaching effect of the two groups of students.

**Results:**

The experimental group was found to be superior to the control group in the quality assessment of complete medical record writing and the ability assessment of diagnosis and analysis of typical obstetrics and gynecology cases (*P* < 0. 05). However, the score of professional knowledge was lower than that of the control group (*P* < 0. 05). The results of questionnaire survey showed that the satisfaction of the experimental group in stimulating learning interest, enhancing problem solving ability, enhancing communication and clinical thinking ability, enhancing team cooperation awareness and independent innovation ability was higher than that of the control group (*P* < 0.01). The satisfaction of teacher-student interaction was also better (*P* < 0.05). However, in terms of strengthening theoretical understanding, the satisfaction of the experimental group was lower than that of the control group, but with no significant difference (*P* > 0.05).

**Conclusion:**

During the epidemic period, we designed a new online teaching mode, which can be applied to the probation teaching of obstetrics and gynecology. In our study, compared with traditional offline teaching, the new online teaching mode could improve students’ ability of case writing and case analysis. However, more teaching practice is needed to complete this online teaching mode.

**Supplementary Information:**

The online version contains supplementary material available at 10.1186/s12909-022-03854-1.

## Introduction

In recent years, with the development of the internet, online teaching has started to become a norm in colleges and universities. However, a lot of online classes are concerned with teaching the extra-curricular knowledge, frontier knowledge, as well as ideological and political education. Most colleges and universities still adopt the offline teaching mode, called lecture-based learning. According to the teachers, classroom teaching continues to be the main teaching mode for non-computer majors, especially the clinical teaching for clinical medicine interns. There are many other forms of offline teaching are in common use,such as small group teaching, peer teaching, PBL and TBL. Because of the interns need to see the patient and make a face-to-face conversation, small group teaching mode is the most commonly used. With the gradual advancements in education and teaching reform from simple offline teaching to online teaching and then to a hybrid model consisting of a mixture of both these modes, clinical teaching is also facing great changes and challenges. The discipline of obstetrics and gynecology involves women’s privacy, patients are often reluctant to cooperate with clinical teaching during clinical probation, and the opportunities for students to observe and practice are significantly reduced. So there is a need for alternative forms of teaching, even in the absence of a pandemic. All the clinical medicine interns of 2020 grade of The First Affiliated Hospital of The University of South China were taught online at home during the COVID-19 pandemic. We constructed a clinical basic integrated teaching model combining case studies and the MOOC platform, after designing, deducting, modifying, and simulating, which was successfully introduced into the clinical internship model of obstetrics and gynecology. As the epidemic had been gradually controlled in China, the traditional novitiate teaching mode was restored in our university. That means the clinical medicine interns of 2021 grade was taken the offline courses in the school. The teaching effect between the two grades was evaluated by the means of a comprehensive test and a questionnaire survey to find and improve the teaching method of probation under the new mode. The purpose of this paper is to discuss the advantages and disadvantages of this new online teaching mode, and to improve its methodology to better serve the specific implementation of online teaching.

## Data and methods

### General information

We selected the 110 clinical interns of grade 2020 of the First Affiliated Hospital of The University of South China as the experimental group, who were asked not to come to school to avoid contracting COVID-19. The experimental group accepted the new online teaching mode combining case studies and the MOOC platform from February to July 2020. The 110 clinical interns of grade 2021 were selected as the control group, who accepted the traditional internship face-to-face teaching mode from February to July 2021, when the epidemic had been gradually brought under control in China. There were 59 men and 51 women in the experimental group, with an average age of 22.21 ± 0.38 years, and 57 men and 53 women in the control group, with an average age was 21.96 ± 0.79 years. There was no significant difference in the age, gender, education background, and other general parameters between the two groups (*P* > 0.05). And no one had contracted the COVID-19 in both groups.

### Methods

The interns of both the experimental group and the control group were randomly assigned to 10 squads at the beginning of the semester, with 11 students in each squad. There were 10 course contents throughout the semester. Each squad had the same course contents. Each squad took the class in turn, one group would be taught each day, and the class period was about 180 minutes. The specific course content was set according to the requirements of the teaching syllabus.

**Fig. 1:**
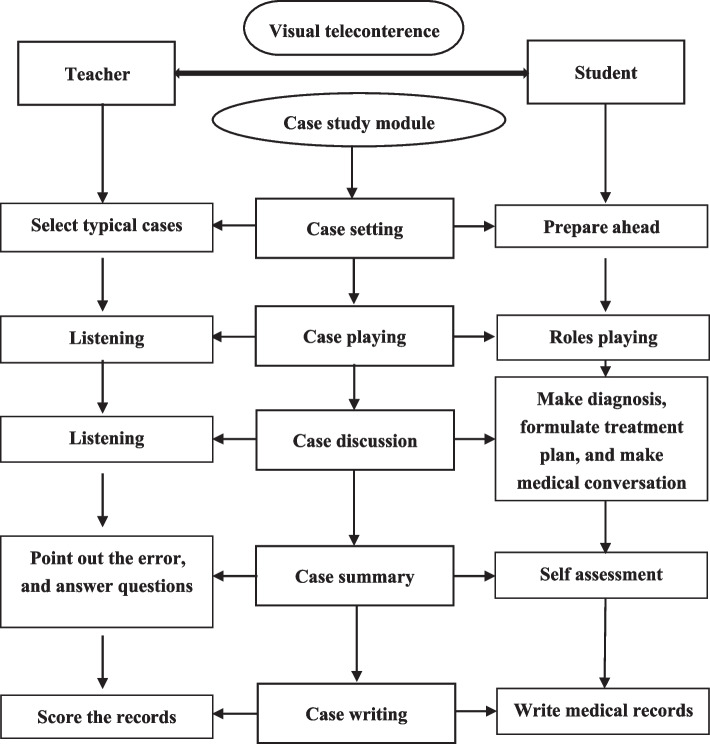
Online case study teaching module

The experimental group adopted the online teaching mode combining online case studies and the MOOC platform. The online students took the visual teleconference (Tencent Conference) as the media and were introduced to the course materials on the MOOC platform. The students first learned the basic theoretical knowledge of diseases, then entered the teaching module of case studies of corresponding diseases, and finally returned to the theoretical summary. The specific implementation plan of the online case study teaching module has been outlined as follows (Fig. 1).

#### (1) Case setting

The teachers were instructed to determine the course content, select one or two typical cases related to obstetrics and gynecology. One student for each case was selected to play the part of Standardized patient (SP), and three or four students were selected to act as residents to conduct a doctor-patient conversation. The SP could preview the typical cases provided by the teacher in advance, while other members in the squad would be required to prepare the disease-related content. This process of preparing for the classes was to be completed by the students independently.

#### (2) Case playing

After the theoretical learning module of the MOOC platform, the SP selected in advance and the resident doctors played the case roles, and obtained clinical data of patients through a detailed history consultation. This process was the primary component of the online course for novices, which trained the students to collect medical history data.

#### (3) Case discussion

The group members made diagnosis on the basis of the information obtained from the consultation, formulated treatment plan, and had a conversation with the SP about the condition of admission, simulating the clinical diagnosis and treatment processes throughout the whole exercise. This stage was the challenging part of the online course, aiming at cultivating students’ clinical thinking ability and training them in communication.

#### (4) Case summary

The SP and other watching team members first evaluated the integrity of the resident clinical activities and whether they were performed correctly, then the teacher’s evaluation of the students would be supplemented, including the clinical manifestations, diagnosis, differential diagnosis and treatment of the case. Meanwhile the teacher could answer any questions the students might have and, if necessary, introduce new concepts and knowledge to broaden the students’ horizons.

#### (5) Case writing

The team members would be asked to write a medical record for each case to improve students’ ability to write medical records, and the teacher evaluated the medical record writing. This stage could be carried out after class.

The control group adopted the traditional teaching mode, which was taught collectively by the teachers according to the syllabus, the students simulated medical work, such as following the clinical team teacher to check the patient and observing how the teacher asking questions about patient’s symptoms and past health.

### Implementation effect evaluation scheme

At the end of the internship, the students were tested on basic professional knowledge, the ability to write complete medical records and their quality, and the ability to diagnose and analyze typical obstetrics and gynecology cases, with a total score of 100 points for each.

For an inspection of the students’ grasp of theoretical knowledge, a questionnaire consisting of 50 questions with 2 points for each question was evaluated by deputy senior teachers from the department of obstetrics and gynecology using a unified grading system. Students were required to complete 10 complete medical records during probation, with one copy for each course content. The last two complete medical records of each student during the probation period were scored by the teacher and their average score was taken as the score of medical record writing to investigate the students’ writing ability. Typical cases of obstetrics and gynecology were selected as case analysis questions to investigate the clinical thinking and case analysis ability of the students.

On the basis of the previous questionnaire survey [1], the study made some improvements to the questionnaire which could be seen in the Appendix 1. The teaching methods were evaluated by more factors, including the degree of teacher-student interaction, strengthening the understanding of theoretical knowledge or not, reinforcing the ability of solving communication and understanding or not, enhancing the sense of teamwork or not, and intensifying the ability of independent innovation or not. The students’ feedback on the teaching model and its advantages and disadvantages were collected through an online questionnaire survey at the end of the semester.

### Statistical methods

The data collected were entered into a Microsoft Excel spreadsheet and statistically analyzed (IBM SPSS Version 26; International Business Machine, Armonk, NY, United States of America). Measurement data were expressed as x ± s. X is the mean and s is the standard deviation. Two independent sample T tests were used for comparison between groups. Counting data were expressed as percentages, and the chi-square test was adopted for the same. *P* < 0.05 was considered statistically significant.

## Results

### Assessment

The experimental group was found to be superior to the control group in the quality assessment of complete medical record writing and the ability assessment of diagnosis and analysis of typical obstetrics and gynecology cases (*P* < 0. 05). However, the score of professional knowledge was lower than that of the control group (*P* < 0. 05), as recorded in Table 1.

**Table 1:** Comparison of scores between experimental group and control group (x ± s, points)

Assessment	Experimental group (***n*** = 110)	Control group (***n*** = 110)	***P*** value
Theoretical examination	79.9 ± 4.4	81.9 ± 4.6	0.00348
Medical record writing	81.9 ± 4.5	80.2 ± 4.9	0.03970
Case analysis	84.0 ± 6.0	81.6 ± 5.5	0.00584

### Interns’ evaluation of the teaching model effect

After the final examination, a total of 220 questionnaires were distributed to all the probationary students, with a recovery rate of 100%. All of them were valid questionnaires. The results of the experimental group in the evaluation of the teaching mode to stimulate interest in learning, strengthen problem solving ability, enhance communication and clinical thinking ability, strengthen the consciousness of team cooperation and the capacity for independent innovation satisfaction were higher than that in the control group; differences were significant statistical significance (*P* < 0.01). The satisfaction degree of teacher-student interaction in the experimental group was higher than that in the control group, and the difference was again statistically significant (*P* < 0. 05). In terms of strengthening theoretical understanding, the satisfaction of the experimental group was lower than that of the control group, but the difference was not statistically significant (*P* > 0. 05). These results have been tabulated in Table 2.

**Table 2:** Comparison of recognition of teaching Methods (n)

Evaluation	Experimental group (***n*** = 110)	Control group (***n*** = 110)	x^**2**^	***P*** value
**Improve interest in learning**			11.579	0.0007
Satisfaction	110	99		
Dissatisfaction	0	11		
**Strengthen theoretical understanding**			2.43	0.1190
Satisfaction	99	105		
Dissatisfaction	11	5		
**Teacher-student interaction**			0.531	0.021
Satisfaction	103	110		
Dissatisfaction	7	0		
**Improve problem-solving skills**			14.08	0.0001
Satisfaction	108	92		
Dissatisfaction	2	18		
**Enhance communication and understanding**			13.82	0.0002
Satisfaction	110	97		
Dissatisfaction	0	13		
**Enhance clinical thinking ability**			8.93	0.0028
Satisfaction	103	88		
Dissatisfaction	7	22		
**Enhance the sense of teamwork**			11.16	0.0008
Satisfaction	105	89		
Dissatisfaction	5	21		
**Enhance our capacity for independent innovation**			8.89	0.0028
Satisfaction	106	93		
Dissatisfaction	4	17		

## Discussion

Clinical probation is very important in the growth process of medical students. It is a preliminary practice for medical students in the theoretical stage of clinical disciplines to consolidate the theoretical knowledge they have gained. It is the key medium for the integration of theory and practice, and an important bridge for the transition from medical students to interns. Traditional probation teaching is given priority, which consists of teachers actively imparting knowledge and the students passively accepting the information [2]. Watching the teacher during the diagnosis and treatment stages is complementary, by teaching the students beside the patients’ bed to the students by means of interpretation of the infusion patient related theory knowledge. A lack of interaction between teachers and students reduces independent thinking, restricting the full mobilization of the students’ enthusiasm for their course of study. This is not conducive for the development of students’ clinical thinking and communication abilities.

‘Obstetrics and Gynecology’ is a required clinical specialty course for medical students in colleges and universities. It is a highly practical subject. As one of the four main disciplines of clinical medicine, obstetrics and gynecology is a specialized subject studying physiological and pathological changes of the female reproductive system and fertility regulation [3]. This course of study is primarily concerned with the prevention and treatment of female reproductive organ diseases as its main teaching and research content, and occupies a large proportion of teaching in higher medical education. However, because the discipline of obstetrics and gynecology involves women’s privacy, patients are often reluctant to cooperate with clinical teaching during clinical probation, and the opportunities for students to observe and practice are significantly reduced. Meanwhile, the family’s protection of female patients also increases the difficulty of medical activities for the trainee. So there is a need for alternative forms of teaching, even in the absence of a pandemic.

In the COVID-19 period of 2020, when classes were suspended, the online teaching mode stands out among the numerous means of teaching reform and becomes the only way of teaching in colleges and universities during the pandemic. With the continuous popularity of internet, online teaching has become a norm in clinical practice teaching [4]. This study aimed to determine which online teaching mode is suitable for the teaching needs of clinical interns in obstetrics and gynecology, and to achieve better teaching effects on the basis of basic teaching objectives.

With the rapid development of information technology, the course teaching mode of the MOOC platform has been widely used [5]. This mode can break through the limitations of time and place and better meet the learning needs of college students. However, this platform still poses considerable problems. First, there is little interaction between teachers and students. Teachers cannot intuitively understand students’ learning progress through the analysis of their facial expressions and language, and their grasp of the class response is poor. The communication between teachers and students is the soul of teaching. Second, students do not have a good learning atmosphere. A large part of the role of a classroom is that it enables the students to study together, and influence and assist each other. For many students, there is no sense of duty and they cannot consciously and effectively complete theoretical learning via the online mode. Thirdly, it is not able to teach students in accordance with their aptitude. MOOC courses are the basic unit to achieve professional graduation requirements, but for medical interns, basic theory learning is far from enough. The internship courses require students to get close to and delve deep into clinical practice.

In the clinical practice classes, the main teaching mode is group teaching, and they are more flexible and diversified. These are the first steps for medical students in their transition to become doctors, and these classes enable them to lay a solid foundation of basic theory and operation skills and cultivate correct clinical thinking, which plays an indispensable role in medical practice. Case study teaching can provide training for students’ subjective initiative in learning, mobilize learning enthusiasm [6], and cultivate communication skills as well as their ability to conclude and summarize [7–9], and also improve students’ learning efficiency [10–12].

In the new teaching mode we set up, case study teaching required students to conduct independent after-class learning according to the teaching content and key points specified by the teacher before class. Because of previewing and consulting materials in advance, students’ enthusiasm for learning could be significantly improved, and their ability of previewing theoretical knowledge and consulting materials could be cultivated as well. Because of the psychology of exploring unknown things, students often had a strong interest in case simulation. Before case simulation, the teacher would help students review the theoretical knowledge again with the help of the MOOC platform, and put forward the key points and difficulties to deepen their understanding. By acting as SP, students could not only stand in the shoes of the patients and experience their feelings but also understand the occurrence and performance of diseases through the process of imitation. The students acting as the visiting doctors were asked to communicate with the SP and discuss the appropriate diagnosis and treatment plan through division of labor and cooperation. After the play, the two sides evaluated each other. In this process, the students’ sense of experience and participation was stronger than the traditional face-to-face teaching, and the students’ enthusiasm significantly improved. In the teaching process of case studies, such role-playing exercises, group case consultation simulation and case discussion were carried out, and the group’s plans were analyzed and summarized, so that the classroom became a place for teachers and students to interact in ways which included discussion and reporting, answering questions and solving doubts [13], formed an active, mutually supportive and problem-oriented learning mode. This facilitated the students’ sense of experience and strong satisfaction in case discussion and research. Meanwhile, teachers could create a positive atmosphere for discussion through case studies and generate a teaching presence. Through in-depth participation and applying theoretical knowledge to the case, students learned about the occurrence and progress of diseases, and then combined the theory and observation for further diagnosis and treatment, which eventually improved students’ clinical thinking and communication ability to make up for the lack generated by the MOOC courses. This teaching method also exercised the students’ ability to organize and express related content, cultivates their ability to communicate with each other, promoted understanding, mastery and application of relevant knowledge, improved teaching quality and cultivated high-quality medical talents.

Case study teaching required teachers to select corresponding representatives, typical, targeted and authentic clinical cases according to the teaching progress, comprehensively analyze and summarize the clinical data, and complete the teaching of writing typical cases [12], which could not only reduce the difficulty of knowledge acquisition for students, but could also effectively cultivate the ability of students to deal with common diseases of obstetrics and gynecology so as to ensure the unity of teaching and effectively improved the teaching quality [14]. At the same time, it also cultivated teachers’ learning ability, improves their teaching level, which solved the problem of teaching effect via the online mode. In recent years, with the developments in higher education, especially in Europe, the United States, and other developed countries, the application of case study teaching is becoming increasingly common, and many studies had demonstrated that class participation caused the students to develop the attitude, habits and methods of self-managing learning behavior and enhanced the students’ learning initiative [15–17].

In this study, by comparing the teaching method of combining case studies and the MOOC platform in probation classes with traditional probation teaching, the assessment results showed that the quality assessment of complete medical record writing and the ability assessment of diagnosis and analysis of typical obstetrics and gynecology cases were better than the control group, but the score of professional basic knowledge was lower than that of the control group. The reason might be that the students of grade 2020 completed the major theoretical courses on the internet by themselves through the teaching videos on the MOOC platform during COVID-19, lacking the sense of participation, teacher’s supervision and the atmosphere of studying together with other students, which affected the completion of students’ theoretical learning to some extent. This is consistent with the idea of Liu Xinli and Zhang Li et al. [18, 19]

According to the results of the questionnaire survey, the experimental group was superior to the control group in terms of satisfactory teaching methods, improved the interest in obstetrics and gynecology, and the ability of teamwork, language expression, analyzing and solving problems, clinical thinking, literature retrieval, and independent learning. This showed that the teacher should create a variety of forms of teaching activities by enriching the teaching resources, such as the preparation before class, roles playing, class discussion, after-school case writing, etc., which helped the student to complete the study in the relaxed and happy classroom atmosphere, aroused the students’ interest in learning, enhanced the interaction with the exchange of students and teachers, and developed better clinical ability capacity. This is similar to the conclusion of Feng Rong et al. [20] However, the questionnaire still had some limitations. For instance, respondents could only reply yes or no, which result in a limit to the information gathered and not further subdivide the real feelings of students. In addition, we should pose the question in a more open way, so that respondents would be able to reply with free text which allowing them to express their own ideas and opinions. These limitations need attention and improvement in future research.

This study was tested only in a very specific scenario with a small group of students from one university, the time period was short, the number of the subject was small. Moreover, there are certain differences from year to year, with younger students being relatively more receptive to online instruction because they may have been exposed to it since childhood, so more practice is still required to further improve and perfect the teaching plan, this can be accomplished through optimization of teaching schemes, reforms in the online teaching mode, conducting the personalized teaching, improve the quality of online probation teaching of obstetrics and gynecology.

In conclusion, during the epidemic period, we designed a new online teaching mode, which can be applied to the probation teaching of obstetrics and gynecology. In our study, compared with traditional offline teaching, the new online teaching mode could improve students’ ability of case writing and case analysis. However, more teaching practice is needed to complete this online teaching mode.

## Supplementary Information

**Additional file 1.**

## Data Availability

These data used to support the findings of this study are available from the corresponding author upon request.
