# Supplementary material for: Application of online teaching mode combining case studies and the MOOC platform in obstetrics and gynecology probation teaching
Source: BMC Med Educ. 2022 Nov 17;22:800. doi: 10.1186/s12909-022-03854-1 (PMC9670045; doi:10.1186/s12909-022-03854-1)
Supplement: Supplementary file 1 — Additional file 1. [file 12909_2022_3854_MOESM1_ESM.docx]

Questionnaire

Male: Age: grade:

In order to improve the quality of teaching, we have designed this questionnaire. This questionnaire is conducted anonymously. Please answer it carefully and objectively, and please mark” √” after your choice.

1. Can probation teaching in this semester improve learning interest and strengthen self-directed learning?

Yes No

1. Can probation teaching in this semester strengthen the understanding of theoretical knowledge and facilitate the assessment of theoretical knowledge?

Yes No

1. Is the probation teaching beneficial to teacher-student interaction?

Yes No

1. Can the probation teaching in this semester enhance the ability to solve learning problems?

Yes No

1. Can the probation teaching in this semester enhance the ability of doctor-patient communication and understanding?

Yes No

1. Can the probation teaching in this semester enhance the clinical thinking ability?

Yes No

1. Can the probation teaching in this semester enhance the awareness of medical team cooperation?

Yes No

1. Can the probation teaching in this semester enhance the ability of independent innovation?

Yes No

1. What do you think are the disadvantages of online teaching? (Optional)

Online courses lack peer learning and learning atmosphere;

There are few opportunities for online courses and face-to-face communication with teachers, and some learning problems are not solved in time；

Online courses lack the supervision of teachers and mutual supervision between classmates, which is easy to be distracted and the learning effect is not good；

Online courses lack of direct visible competition and contrast, lack of learning motivation, low initiative；

There is no penalty mechanism for online courses, and homework is sloppy, resulting in poor learning；

The assessment method of online courses is not adapted, and the learning results can not be displayed；

Others,like:

**Which teaching method do you prefer?**

Online teaching

Offline teaching

combination of online and offline.
